# Exploring the Mechanisms for Virus Invasion at the Barrier of Host Defense Involving Signaling Pathways

**DOI:** 10.3390/v16121939

**Published:** 2024-12-19

**Authors:** Bumsuk Hahm

**Affiliations:** Departments of Surgery & Molecular Microbiology and Immunology, University of Missouri, Columbia, MO 65212, USA; hahmb@health.missouri.edu

Pathogenic viruses trigger or disrupt multiple signaling networks to establish an environment optimized for their own replication and productive infection. Understanding the core mechanisms of virus–host defense interactions and viral pathogenesis could lead to innovative therapeutics for viral diseases. The Special Issue “Viral Strategies to Regulate Host Immunity or Signal Pathways” covered research topics such as virus interaction with the host innate and adaptive immunity and the viral regulation of intracellular signaling pathways.

In a research article by Zhang et al. [1], herpes simplex virus type 2 (HSV-2) was shown to use viral UL24 to strongly impair IRF3 phosphorylation and suppress the subsequent synthesis of type I interferons (IFNs). The study further revealed that the N terminal 1–202 AA domain of UL24 displayed inhibitory activity that could overcome the activity of IRF-3 or its upstream molecule-induced IFN production. As the type I IFN is the most potent innate immune molecule against virus infections, numerous pathogenic viruses in humans seem to have developed ways to attenuate the IFN response [2,3]. As such, extensive research has been performed to identify cellular proteins of the IFN system that can be targeted for designing new host-directed therapies to combat viral infections. The study by Yu et al. [4] investigated the function of Naringenin, which is a flavonoid found in citrus fruits with diverse pharmacological activities linked to the host innate immunity to porcine reproductive and respiratory syndrome virus (PRRSV) infection. Interestingly, Naringenin enhanced the expression of antiviral cytokines such as type I IFNs and IFN-stimulated genes that were suppressed by PRRSV. The study indicated that Naringenin could activate the RIG-I-MAVS signal pathway. Since PRRSV is known to induce immune suppression, the use of an immune stimulatory agent such as Naringenin may help to prevent the syndrome caused by PPRSV infection. However, any immune regulatory agent needs to be investigated for the possible induction of harmful inflammation and consequent tissue damage. Indeed, the type I IFN response can become pathogenic to the host. The protective- or pathogenic-type I IFN responses have been reviewed in an article written by Jung et al. [5]. Type I IFNs were first observed to interfere with influenza virus replication by Isaacs and Lindenmann in 1957 [6], and have been therapeutically used to inhibit multiple viruses and immune-mediated diseases such as multiple sclerosis. However, they could also cause inflammation or support T cell exhaustion in certain chronic viral infections [7,8], requiring in-depth interrogation of their mode of action. Type I IFNs have versatile functions that could be affected by factors including the timing of IFN production, type of IFN-responsive cells or pathogens, the stage of immune cell differentiation, etc. Further research in this area remains to be conducted.

Neutrophils are important innate immune cells that contribute to the clearance of diverse pathogens as well as the induction of inflammatory reactions via the secretion of toxic molecules through granule exocytosis. Lei Hongxing performed transcriptome studies using the blood of patients infected with SARS-CoV-2, from which the most prominent functional category of upregulated genes was identified as neutrophil degranulation and cell cycling [9]. Further, both physiological and pathological hypoxia can induce the action of genes related to neutrophil degranulation, which further necessitates a comprehensive analysis of the roles of hypoxia [10] and neutrophil responses during COVID-19.

Additionally, the role of ferroptosis during viral pathogenesis was reviewed by Huang et al. [11]. Ferroptosis is a type of cell death caused by intracellular iron metabolism that regulates reactive oxygen species (ROS) and mediates lipid-dependent oxidative damage [12]. Viruses have often been reported to manipulate cellular ferroptosis to promote viral proliferation. Various steps of viral replication processes or immune evasive mechanisms have been shown to be affected by viral regulation of ferroptosis: examples include regulation of the transport pathway of TfR1 for effective viral entry, increased iron levels for promoting viral enzyme function, and the accumulation of lipid peroxides for viral immune escape.

In summary, these studies indicate that it is imperative to uncover the mechanisms of virus–host interplay and the viral regulation of cellular pathways. A better understanding of the host defense mechanism as well as viral tactics to avoid host immunity should help design advanced therapeutic approaches and improve human health.
